# Supplementary material for: The contribution of birth plans to shared decision-making from the perspectives of women, their partners and their healthcare providers
Source: PLoS One. 2024 Jun 26;19(6):e0305226. doi: 10.1371/journal.pone.0305226 (PMC11207161; doi:10.1371/journal.pone.0305226)
Supplement: S1 Table — (DOCX) [file pone.0305226.s001.docx]

**S1 Table. Topic list interviews with women**

| Topics | Initial questions | Extra questions/topics |
| --- | --- | --- |
| Role of birth plan/ process of use | - Grand tour question: Take me back to when the birth plan came into being. How did it go? | - How introduced? - Reason for use? - How was the birth plan discussed? - Contribution of healthcare provider? - Contribution of partner? |
| Implementation of the birth plan / role of healthcare provider | - Was the birth plan fully complied afterwards? - What was your healthcare provider's role in the birth plan? | - How did you experience the care during labor? - Were your wishes listened carefully? - Did you receive advice in making the birth plan? - How did the communication go if the labor did not go as planned? |
| Relation with shared decision making | - Are you familiar with the term shared decision making? Can you explain in your own words what you think this term means? - What do you think is the contribution of a birth plan with regard to shared decision making with a healthcare provider? | - What are important factors that can influence shared decision making? - How did your birth plan contributed in these factors? - Was there shared decision making during your pregnancy or labor? - Facilitators and barriers |
| Experiences birth plan after labor | - How did you experience the birth plan afterwards? | - Advantages and disadvantages of using a birth plan. - Future: would you use a birth plan again for a possible next pregnancy? Would you do something different? - How can healthcare providers contribute to possible better care? - What could healthcare providers have done better in your case? |
