# Supplementary material for: The contribution of birth plans to shared decision-making from the perspectives of women, their partners and their healthcare providers
Source: PLoS One. 2024 Jun 26;19(6):e0305226. doi: 10.1371/journal.pone.0305226 (PMC11207161; doi:10.1371/journal.pone.0305226)
Supplement: S2 Table — (DOCX) [file pone.0305226.s002.docx]

**S2 Table. Topic list interviews with partners**

| **Topics** | **Initial questions** | **Extra questions/topics** |
| --- | --- | --- |
| **Experience of childbirth** | - Grand tour question: Could you describe the childbirth, how did it went down? | - How did you, as a partner, experience the child delivery? - Which role did you take on during the delivery? - How did it feel to fulfill this role? - How did you experience the preparation of the delivery? - How has your opinion influenced choices you and your partner made? - How did you experience the communication with the care providers? - To what extent has the care provider involved you in the decisions regarding the delivery? |
| **Role of the birth plan** | - Did you draw up a birth plan before the delivery? - Take me back to when the birth plan came to be, how did this go? | - What was the reason you used a birth plan? - Do you have multiple children? If yes, did you also use the birth plan during a previous delivery? - What did the birth plan consist of? - What role did the birth plan have during the delivery? - To what extent had the use of a birth plan increased your involvement, as a partner? |
| **Implementation of the birth plan** | - To what extent had the birth plan been complied with? |  |
| **Experience birth plan after child birth** | - How did you experience the birth plan, afterwards? | - What do you think are the disadvantages of using a birth plan? - What do you think are the advantages of using a birth plan? - Would you use a birth plan again, for a possible next pregnancy? - What would you do differently next time with regard to the birth plan? |
| **Role healthcare provider regarding birth plan** | - To what extent has your healthcare provider contributed to drawing up of the birth plan? | - To what extent has the healthcare provider involved you, as a partner, in drawing up the birth plan? - To what extent has your healthcare provider contributed to complying the birth plan? |
| **Relationship birth plan and SDM** | - Can you tell me what shared decision-making is? | - What do you think is the contribution of birth plans in the process of SDM? |
