# Supplementary material for: The contribution of birth plans to shared decision-making from the perspectives of women, their partners and their healthcare providers
Source: PLoS One. 2024 Jun 26;19(6):e0305226. doi: 10.1371/journal.pone.0305226 (PMC11207161; doi:10.1371/journal.pone.0305226)
Supplement: S3 Table — (DOCX) [file pone.0305226.s003.docx]

**S3 Table. Topic list interview healthcare providers**

| **Topics** | **Initial questions** | **Extra questions/topics** |
| --- | --- | --- |
| **Introduction** | - Where do you work? - What is your function? - How many years have you been working here? | - Birth plan: Are you familiar with birth plans. What does this mean? - SDM: Are you familiar with shared decision-making? What does this mean? |
| Introduction birth plan/ role birth plan | - How did you experience the introduction of birth plans? | - How were birth plans introduced at the time in your department? - Why are birth pans introduced? - Has this been discussed with your department at the time? - Are patients being advised in making a birth plan? |
| Implementation in pregnancy | - How are birth plans implemented during pregnancy? | - What is the role of women in creating a birth plan? - What is the role of partners in creating a birth plan? - What is the role of healthcare provider in creating a birth plan? |
| Implementation of the birth plan during childbirth | - How are birth plans implemented during childbirth ? | - What is the role of women regarding a birth plan? - What is the role of partners regarding a birth plan? - What is the role of healthcare provider regarding a birth plan? |
| Experience with implementation of birth plan | - How do you experience the implementation of the birth plan? | - How do you experience patient care during childbirth with the use of a birth plan? - Are the wishes of women often completely fulfilled? - What if wishes can not be fulfilled? - What if patients have unrealistic wishes? |
| Effect birth plans on shared decision-making | - What effect do you think the use of birth plans has on shared decision-making in birth care? | · What effect do you think the use of birth plans has on communication between healthcare provider and women?  · What are the benefits of using a birth plan?  And regarding SDM?  · What are the disadvantages of using a birth plan? And regarding SDM?  · Is there a difference in communication |
