# Supplementary material for: The contribution of birth plans to shared decision-making from the perspectives of women, their partners and their healthcare providers
Source: PLoS One. 2024 Jun 26;19(6):e0305226. doi: 10.1371/journal.pone.0305226 (PMC11207161; doi:10.1371/journal.pone.0305226)
Supplement: S4 Table — (DOCX) [file pone.0305226.s004.docx]

**S4 Table. Characteristics of women participating in the interview study**

| **Participant** | **Age** | **Gestational age at labor** | **Gravida/para** | **Mode of birth** | **Medical indication** |
| --- | --- | --- | --- | --- | --- |
| 1 | 26 | 38w5d | G1P1 | Spontaneous vaginal birth. Other: episiotomy. | Induction of labor for prolonged rupture of membranes |
| 2 | 36 | 37w5d | G1P1 | Spontaneous vaginal birth. Other: manual placenta removal | Pain relief request. |
| 3 | 38 | 40w5d | G1P1 | Spontaneous vaginal birth | Pain relief request and  meconium stained fluid. |
| 4 | 34 | 40w5d | G1P1 | Secondary caesarean section | Breech |
| 5 | 30 | 40w2d | G1P1 | Spontaneous vaginal birth | Pain relief request and prolonged labor |
| 6 | 30 | 38w2d | G3P2 | Spontaneous vaginal birth | History of Caesarean section |
| 7 | 36 | 40w4d | G2P2 | Spontaneous vaginal birth | Meconium- stained fluid. |
| 8 | 38 | 41w5d | G4P2 | Assisted vaginal birth. | History of fetal growth restriction |
| 9 | 33 | 39w2d | G2P2 | Spontaneous vaginal birth. | Congenitally abnormality child and pain relief request. |
| 10 | 32 | 39w2d | G2P2 | Caesarean section | History of Caesarean section. |
