# Supplementary material for: The contribution of birth plans to shared decision-making from the perspectives of women, their partners and their healthcare providers
Source: PLoS One. 2024 Jun 26;19(6):e0305226. doi: 10.1371/journal.pone.0305226 (PMC11207161; doi:10.1371/journal.pone.0305226)
Supplement: S5 Table — (DOCX) [file pone.0305226.s005.docx]

| Participant | Age (of partners) | Childs gestational age at birth | Gravida/  para | Mode of childbirth | Medical indication | Start pregnancy | Start parturition | End parturition |
| --- | --- | --- | --- | --- | --- | --- | --- | --- |
| 1 | 38 | 41w5d | G4P2 | Assisted vaginal birth. Other: vacuum extraction | Fetal growth restriction and induced labor | Primary care | Secondary care | Secondary care |
| 2 | 31 | 38w4d | G1P1 | Spontaneous vaginal birth | Own preference hospital birth/prolonged labor and OR stitching | Primary care | Secondary care | Secondary care |
| 3 | 35 | 39w0d | G4P3 | Spontaneous vaginal birth | History of stillborn and fetal distress | Secondary care | Secondary care | Secondary care |
| 4 | 30 | 40w0d | G1P1 | Spontaneous vaginal birth | Pain relief request and prolonged labor | Primary care | Primary care | Secondary care |
| 5 | 35 | 38w0d | G3P2 | Spontaneous vaginal birth | Fetal growth restriction | Primary care/Secondary care after 30w | Secondary care | Secondary care |
| 6 | 35 | 38w3d | G2P2 | Spontaneous vaginal birth | Breach | Primary care/ Secondary care after 35w | Secondary care | Secondary care |
| 7 | 38 | 40w2d | G2P1 | Assisted vaginal birth. Other: vacuum extraction and episiotomy | Pain relief request and prolonged labor | Primary care | Primary care | Secondary care |
| 8 | 33 | 39w3d | G1P1 | Spontaneous vaginal birth | History of fetal growth restriction | Secondary care | Secondary care | Secondary care |
| 9 | 29 | 40w4d | G1P1 | Secondary section caesarea | Meconium-containing amniotic fluid | Primary care | Secondary care | Secondary care |

**S5 Table. Characteristics of women whose partners were interviewed in the study**
