# Supplementary material for: The contribution of birth plans to shared decision-making from the perspectives of women, their partners and their healthcare providers
Source: PLoS One. 2024 Jun 26;19(6):e0305226. doi: 10.1371/journal.pone.0305226 (PMC11207161; doi:10.1371/journal.pone.0305226)
Supplement: S6 Table — (DOCX) [file pone.0305226.s006.docx]

**S6 Table. Characteristics of healthcare providers participating in the interview study**

| **Participant** | **Function** | **Care** | **Gender** |
| --- | --- | --- | --- |
| 1 | Obstetrician in training | Secondary care | Female |
| 2 | Gynecologist | Secondary care | Female |
| 3 | Gynecologist in training | Secondary care | Female |
| 4 | Gynecologist in training | Secondary care | Female |
| 5 | Midwife | Secondary care | Female |
| 6 | Midwife | Secondary care | Female |
| 7 | Midwife | Secondary care | Female |
| 8 | Midwife | Primary care | Female |
| 9 | Midwife | Primary care | Female |
| 10 | Midwife | Primary care | Female |
| 11 | Midwife | Primary care | Female |
| 12 | Midwife | Primary care | Female |
| 13 | Gynecologist in training | Secondary care | Male |
