# Supplementary material for: The contribution of birth plans to shared decision-making from the perspectives of women, their partners and their healthcare providers
Source: PLoS One. 2024 Jun 26;19(6):e0305226. doi: 10.1371/journal.pone.0305226 (PMC11207161; doi:10.1371/journal.pone.0305226)
Supplement: Attachment — Submitted filename: Response to Reviewers.docx [file pone.0305226.s007.docx]

# **Rebuttal letter**

We would like to express our sincere gratitude for the opportunity to revise our manuscript and for the constructive comments provided by the academic editor and reviewers. We have carefully considered each comment and have made corresponding revisions to the manuscript. Below, we respond to each point raised:

# **Reviewer 1**

Overall Impression

This manuscript describes how birth plans can be used by women, their partners and healthcare providers to facilitate shared decision-making and pertinent communication during pregnancy and birth. Having the perspectives of the pregnant women, of partners and healthcare professionals adds to the understanding of the issue. It also emphasizes important barriers on the use of birth plans from various angles.

The design of the study, sampling strategy and data analysis are appropriate and described in detail. The Themes and Sub-Themes are easy to follow and the quotes are pertinent. The Results contribute to obstetrical knowledge and are transferable to other practice settings.

A few issues could improve the overall understanding of the topic, as well as the readability of the manuscript.

Areas for improvement

1. It would be useful to have one sentence about the Netherlands’ context and how pregnant women are usually followed (primary/secondary follow-up/referrals during pregnancy or birth) as systems vary from one country to another.

**Our response:**

Thank you for your valuable suggestion to include a sentence about the Dutch maternity care system. Understanding the national context is indeed crucial for interpreting our study's findings, given the variance in maternity care systems across countries. We have added the following sentence to provide clarity on how pregnant women are typically followed in the Netherlands (line numbers 71-77):

*In the Netherlands, the maternity care system is known for its physiological approach to pregnancy and childbirth, offering primary care to low-risk women and secondary care and tertiary care for higher-risk pregnancies. This system provides options for home or outpatient hospital births for low-risk women and hospital births for those in secondary care. It is all underpinned by the 2016 Integrated Maternity Care Standard, which recommends individual birth care plans for every pregnant woman[7].*

2. The paragraph in the literature review referring to the aims of a birth plan lacks references.
- A 2022 systematic, integrative review on Birth plans by Bell et al, offers a variety of references that could be used.

**Our response:**

Thank you for your suggestion. We have now added references in lines 86-100 that discuss the various aims of a birth plan.

3. In the Methods paragraph, the following sentence should be clarified to avoid confusion: “We combined data from three interview studies concerning birth plans in relation to SDM from women’s, partners’, and healthcare providers’ points of view.” It could be interpreted as data from 3 different studies were merged, whereas 3 groups of people were interviewed within one study.

**Our response:**

Thank you for pointing out the need for clarification in our Methods section. We have revised the sentence to accurately convey that the interviews with women, their partners, and healthcare providers were part of a single study. The updated sentence (page 6, lines 110-112) is:

*In our research, we combined data from interviews concerning birth plans in relation to SDM with three distinct groups – women, partners, and healthcare providers –, all conducted within the framework of one study.*

4. Some paragraphs could gain readability by making them more concise and ensuring there are no redundant sentences. For example:
a. The paragraph on the Research Team and Reflexivity. Some sentences could be removed.

**Our response:**

We acknowledge your suggestion to enhance readability by making some paragraphs more concise and by removing redundancy. In line with your advice, we have streamlined the paragraph on the Research Team and Reflexivity. The revised paragraph is as follows on page 10, lines 205-208:

*Three Radboud University medical students, NSc, SL, and PS, with initial supervision from JvD, conducted interviews with women, their partners, and healthcare providers, respectively. JvD, NSh, MN, and MdV contributed to article revisions, while JvD, MH, and MN, experts in midwifery/obstetrics and SDM, guided the research.*

b. The grounded theory: birth plans as a tool for SDM. Many points from the Discussion are repeated.

**Our response:**

We retained the grounded theory regarding birth plans as a tool for SDM on page 25-26 lines 526-538, and revised the discussion to eliminate repetitions, as indicated on page 26-28.

c. The discussion itself could be more concise.

**Our response:**

Thank you for your feedback on the discussion section. We've restructured it to enhance conciseness and readability, ensuring the main points are clearly communicated. The revised discussion is presented on pages 26-28:

*This study highlights the effectiveness of birth plans in enhancing SDM between pregnant women, their partners, and healthcare providers, emphasizing their contribution to both internal and external empowerment. Our grounded theory suggests that creating a birth plan encourages expectant women and partners to discuss preferences and gain knowledge about various childbirth scenarios, but it also enhances their ability to engage in effective conversations with each other and with their healthcare provider. Ultimately, this is perceived to lead to well-informed decisions through shared decision-making[8, 14, 20-28].*

*Healthcare providers, women, and their partners all value SDM, acknowledging the birth plan as essential for guiding discussions on childbirth preferences. They concur on the necessity of realistic expectations due to childbirth's unpredictability. However, while women and their partners view the birth plan as a tool for empowerment and autonomy, healthcare providers see it primarily as a means for communication and expectation management. Professionals aim to balance patient desires with medical advisability, ensuring safety and feasible outcomes. In contrast, expectant mothers and partners emphasize personal choices and control, highlighting the complex interplay between individual preferences, medical advice, and the uncertainties of childbirth.*

*Internal empowerment, as characterized by increased knowledge, confidence, and self-advocacy in women engaged in birth planning, is supported by multiple studies[10, 28, 29].* *This empowerment reflects the psychological and emotional strength women gain from actively participating in their birth planning.*

*External empowerment is facilitated by the role of healthcare providers. The importance of healthcare providers' supportive and validating approach in the successful implementation of birth plans has been emphasized in the work of several researchers, highlighting how such support integrates women's preferences into clinical practice[30-32].* *This necessitates clear communication and a deep respect for women's autonomous choices, integrating them seamlessly with the realities of medical practice.*

*The dialogue between healthcare providers and women, focusing on realistic expectations and the unpredictability of childbirth, is a critical component of external empowerment. Multiple studies have also noted the importance of these discussions in aligning expectations with feasible medical practices[21, 26, 31].*

*Further, the study underscores the necessity of ongoing education and communication about birth plans. Multiple studies advocate for antenatal education addressing care provision differences in various settings, assisting women in making informed choices regarding their birthing environment[8. 30, 33].* *This aspect of education is crucial in ensuring that birth plans are both realistic and achievable.*

*Additionally, the recognition and respect for cultural and individual differences in birth plan creation and execution are paramount. This approach, as supported by research from authors such as Welsh et al., ensures that plans are tailored to individual needs, enhancing their effectiveness and contributing to a comprehensive approach to empowerment in SDM[30].*

*In conclusion, birth plans are instrumental in promoting empowerment and successful SDM. Their effectiveness depends on a balanced approach that respects medical guidelines, individual preferences, and cultural nuances, ultimately honoring the autonomy and preferences of the pregnant woman and her partner.*

d. In the paragraph on Strengths and Limitations, the purpose of the lengthy description of the hospital is unclear.

**Our response:**

We appreciate your input on the need for conciseness regarding the detailed description of the hospital in the Strengths and Limitations paragraph. We've revised this section to succinctly reflect on the role of the hospital's specific environment in the study. The modified text is as follows on page 28, lines 596-603:

*There are some limitations to this study. One limitation is that it was conducted solely at Radboudumc. This is a academic teaching hospital providing secondary care for the local community and tertiary care for higher-risk pregnancies for the larger region. This hospital is committed to person-oriented care and the first in the Netherlands to endorse the Salzburg Declaration for shared decision-making. This dedicated environment, already focused on collaborative care with patients, may have influenced the study's outcomes. However, this focus also acts as a strength, providing a unique perspective on the efficacy SDM within a pioneering healthcare setting dedicated to person-oriented and collaborative care.*

5. Some sentences could be reworded for the article to gain readability. Some examples:
a. “Given the importance of SDM and the possible impact of birth plans, research is needed to gain more insight into how this can work.”

**Our response:**

Thank you for your suggestion to improve the readability of the article. We have taken your advice into consideration and reworded the sentence for clarity and conciseness, in lines 102-103. The revised sentence is now as follows:

*Considering the significance of SDM and the potential influence of birth plans, further research is essential to better understand their effectiveness and implementation.*

b. “The interviews brought to the attention that all stakeholders found it important that they talked to each other about their preferences for birth, while at the same time remaining realistic in making decisions about their birth and taking in mind that not all options may always be possible or desirable, because they might consider some risks to be unacceptable for the woman or her baby.”

**Our response:**

The revised sentence is now as follows, in lines 389-392:

*The interviews revealed a consensus among stakeholders about the importance of discussing birth preferences. Simultaneously, they emphasized the need for realism in decision-making, acknowledging that not all options may be viable or advisable due to potential risks for the woman or her baby*

c. “Women expressed the anticipation of having choices”

**Our response:**

The revised sentence is now as follows, in lines 424-225:
*Women highlighted their expectation of having choices and underscored the importance of healthcare providers adopting an approach that is open, impartial, and practical.*

6. Some sentences are incomplete. For example:
a. “The knowledge that certain aspects (such as fulfilling all the preferences outlined in the birth plan, which may be impossible) adds more pressure to their role.”

**Our response:**

The revised sentence is now as follows, in lines 438-440:

*Healthcare providers frequently reported feeling inadequate when dealing with complex and extensive birth plans, as the difficulty in meeting all the specified preferences, some not viable or advisable, added to the pressure of their responsibilities*

Reviewer #2:

I consider the document presents an interesting work that particularly contributes to the research field showing how women, their partners and health providers perceive the contribution of birth plans to shared decision-making (SDM). Methodologically, the document describes in a sufficient and adequate form the process of data collection and analysis. The research results are presented in an orderly manner and are consistent with the methodology used.

According to what the authors and other research have also found, the use of birth plans seems to contribute to SDM. Nevertheless, some difficulties have also been identified. Among the findings of this research, that describes what three groups of stakeholders say about the topic, some characteristics that can contribute to solving the problems have been described.

The document also has some aspects that I consider the authors must improve to clarify, organize, and consolidate the work. By section these are my recommendations:

Introduction
The authors present the Shared Decision-Making (SDM) part in a clear and precise way. The information related to de aims of a birth plan however is not clearly supported. The reference 8 is a study made in women with high-risk pregnancies, and the reference 11 is a link to a Dutch web page where it is not possible to verify the cited information in English.

**Our response:**

Thank you for your suggestion. We have now added references in lines 86-100 that discuss the various aims of a birth plan. We also deleted reference 11 to the Dutch web page and reference 8, a study made in women with high-risk pregnancies.

This section lacks in my opinion information about the context where the research was done. For example, the childbirth care in the Netherlands has some particularities that would be important to mention.

**Our response:**

We added this part to the introduction, lines 71-76:
*In the Netherlands, the maternity care system is known for its physiological approach to pregnancy and childbirth, offering primary care to low-risk women and secondary care and tertiary care for higher-risk pregnancies. This system provides options for home or outpatient hospital births for low-risk women and hospital births for those in secondary care. It is all underpinned by the 2016 Integrated Maternity Care Standard, which recommends individual birth care plans for every pregnant woman[7].*

Methods
This section is one of the strongest parts of the paper.

In this section the authors included data that I consider must be presented in results. For example, in the subtitles Women and partners and Healthcare providers, they present numbers of contacted persons. They also cite tables (S1, S2 and S3) that I supposed will present data of the contacted persons, but instead they present information of the interviewed.

**Our response:**

We acknowledge the confusion caused by the misplacement of data between the sections and the incorrect citation of tables. We realized the initial tables S1, S2, and S3 were meant to display the topic list, but due to a mix-up in the order, this was not accurately represented. We have now corrected this issue, ensuring the tables correctly reflect the intended information. The tables initially referred to as S1, S2, and S3, which were supposed to present data on the contacted persons but instead detailed the interviewed participants, have now been correctly renumbered as S4, S5, and S6 to reflect the characteristics of the participants. We have moved these tables **to page 11** in the Results section. Additionally, we have clarified in line 215-217 that tables S4, S5, and S6 (previously S1, S2, and S3) contain information about the interviewed.

Besides, the title of Tables S2 is not precise; most of the information included in this table it´s not related to partners.

**Our response:**

Thank you for highlighting the lack of precision in the title of Table S2(now table S5). After reevaluating the table, we acknowledge that the data primarily describe the characteristics of women whose partners have been interviewed for our study, rather than the partners themselves. Therefore, we have updated the title of Table S5 to accurately convey the content of the data. The revised title is: 'S5 Table. Characteristics of Women Whose Partners Were Interviewed in the Study.' This modification ensures the title accurately reflects that the table details the characteristics of the women related to the interviewed partners. Additionally, we note that the only characteristic of the men we have recorded is their age. Further characteristics of the partners are not mentioned, as they were not relevant to our research question.

The authors also insert the Figure 1 in the methods section, but the categories it presents are a result of their analysis.

**Our response:**

We have acknowledged the concern regarding the placement of Figure 1 **(now figure 3, page 11,** because of additional figures) in the methods section. Considering that the categories presented in the figure are indeed outcomes of our analysis, we have now appropriately relocated Figure 1 (now Figure 3) to the analysis section of the paper

In the first part of this section the information about the institution where the research was done is missing; some information is presented in the Strengths & limitations section. Besides it would give the readers a better perspective, it would contribute to understand the section 3.1 of Results, which is directly related to some of the characteristics of the institution.

**Our response:**

Addressing the feedback received, we have revised the initial part of the Methods section to include information about the institution where the research was conducted, as following (page 6, lines 112-117):

*These studies were performed at the Radboud University Medical Center (Radboudumc) in Nijmegen, the Netherlands. This is a academic teaching hospital providing secondary care for the local community and tertiary care for higher-risk pregnancies for the larger region. This hospital is committed to person-oriented care and the first in the Netherlands to endorse the Salzburg Declaration for shared decision-making.*

I also recommend the authors to explain with more detail the process and the criteria based on which the participants have been chosen. For example, it´s not clearly described how they select the interviewed among the approached; neither which is the relationship between each women and the partners and health personal interviewed.

**Our response:**

We appreciate your recommendation and have updated our manuscript to provide a more detailed explanation of the participant selection process, on pages 7-8). We have clarified how the interviewees were selected from those who were approached, and we have also elaborated on the relationships between the interviewed women, their partners, and the healthcare personnel involved in the study. Additionally, we have created a flowchart that visually represents the selection process (Fig.1 and Fig. 2)

Results
I consider this section the best part of the paper.
In the first part of it, I recommend the authors to present a more detailed view of the general characteristics of the participants.

**Our response:**

In response to the recommendation for presenting a more detailed view of the participants' general characteristics, we have opted for providing broader characteristics, especially regarding healthcare providers, due to privacy concerns. This decision was made because in the Nijmegen region, many providers are well-acquainted with each other, and we aim to preserve their anonymity. This consideration has also been addressed in the manuscript, lines 157-161, to ensure clarity on our approach regarding privacy and the level of detail provided about participant characteristics.

There is an error in the number of the tables cited at the end of the first paragraph (the correct ones are S1, S2 and S3).

**Our response:**
Thank you for pointing out the discrepancy in the table numbers cited at the end of the first paragraph. The tables were indeed mixed up. We have now corrected the order: in the Methods section, the tables are referred to as S1, S2, and S3 (page 9, displaying the topic list of the participants. In the Results section, the tables have been changed to S4, S5, and S6, which detail the characteristics of the participants (page 11).

Discussion
As the authors highlight, I also consider the principal contribution of their work is the triple perspective (women, their partners and, health providers) of the research topic, but the discussion is focused on the findings of each group in a separate form. I consider the authors should highlight the similarities and differences in the way each group perceives the problem and a how they can complement or contradict each other. This analysis can contribute to identify and possibly help, to resolve the problems described in the use of birth plans in SDM.

**Our response:**
Thank you for your feedback. In response, we've incorporated a concise analysis on page 26, lines 548-556) highlighting the shared and distinct perspectives of women, their partners, and healthcare providers on the use of birth plans in SDM. This section emphasizes the unanimous agreement on the importance of SDM and the necessity for realistic expectations given childbirth's unpredictability. It also outlines how birth plans are viewed differently: as empowerment tools by women and partners, and as aids for communication and expectation management by healthcare providers. This addition seeks to clarify the discussion by explaining the complex relationship between personal choices, medical advice, and the uncertainties of childbirth, directly addressing the challenges and opportunities of using birth plans in the SDM process.

References
I found many errors and inconsistencies in this section.

On page 31 a paper from Fernandez-Arroyo is cited, but it is not included in the references.

**Our response:**

Thank you for bringing this to our attention. Upon review, we have removed the citation of the paper by Fernandez-Arroyo from page 31, as it was not available in English and therefore not accessible to all readers.

The author´s last name cited in page 33 (Farahat) is incorrect.

**Our response:**

We have changed the author's name on page 33 from 'Farahat' to 'Ahmadpour'.

The reference related to COREQ (25), it is not included in the final references.

**Our response:**

We included the reference related to COREQ on page 10, line 201.

In this references the central topic discussed in the cited paper is not clearly related to the topic discussed by the authors: 15 do not assess birth plans; 19 is about women’s motivations for choosing a high-risk birth setting; 20 is about traumatic childbirth and birth plans were no assessed; 24 is a research on the use of birth plans in Life-Limiting Fetal Diagnoses; 27 is a study to validate the reliability of a Farsi version of a scale use to measure different aspects of childbirth fear; 29 is a paper about the concept of a woman's empowerment in pregnancy, childbirth and the postnatal period; 30 is a research of midwives perceptions of their role within the context of maternity service reform.

**Our response:**

Thank you for your feedback. We have removed the referenced sources that were not directly relevant to our discussion on birth plans (reference 15,18,20,24,27,30). Additionally:

- For source 29 (now 32), I utilized it to outline what is required for external empowerment, according to the study. This is apart from the birth plan, focusing on what is generally needed for empowerment. We believe if I clarify this use and adjust the context accordingly, we can retain this reference effectively.

Reference 26 is not traceable. References 36 and 37 are web pages in Dutch. It is not possible to verify the cited information in English.

**Our response:**

We removed these references.

Strengths & limitations
The authors should explain better the reasons why the Radboudumc care characteristics are considered a limitation for the study. Also, I consider the women recall bias can influence the results not only in the “more thought and meaning” way, but also in the opposite way.

**Our response:**

Thank you for your insightful feedback regarding the potential impact of recall bias in our study. We agree that recall bias can influence the results not only by leading participants to give more thought and meaning to their experiences but also in the opposite way, where details might be forgotten or diminished over time. We have revised the relevant section of our manuscript to reflect this broader understanding of recall bias. The updated text (page 28-29, lines 604-609) now reads:

*"**Secondly, the potential for recall bias should be considered, as the women, partners, and healthcare providers were interviewed one to six months postpartum. This time gap might have influenced their recollections, leading them to either ascribe more thought and meaning to their experiences or, conversely, to forget or downplay certain aspects of their experiences and decisions. This dual nature of recall bias could have impacted the results in varying ways."*

The form that the authors use to describe how the findings of other studies are related to their own findings frequently is not clear. For example, they present their findings and then insert some citation numbers, without any comments.

**Our response:**

In response to your feedback, we have revised the discussion section, as previously mentioned, and have taken into account the need for clearer connections between our findings and those of other studies. We've ensured to comment directly on how these citations relate to our research, enhancing the clarity of how our findings contribute to the existing body of knowledge.
