# Supplementary material for: The contribution of birth plans to shared decision-making from the perspectives of women, their partners and their healthcare providers
Source: PLoS One. 2024 Jun 26;19(6):e0305226. doi: 10.1371/journal.pone.0305226 (PMC11207161; doi:10.1371/journal.pone.0305226)
Supplement: Attachment — Submitted filename: Response to reviewer.docx [file pone.0305226.s008.docx]

# Response to reviewer

Dear Dr. Ivan Sarmiento,

We sincerely thank you and the reviewer for carefully reading our manuscript and for the constructive feedback provided. We appreciate the time and effort you took to review our work.

We have made all the suggested minor revisions, primarily grammatical, in accordance with your recommendations. We have clearly indicated each correction and modification in the attached document using track changes to facilitate your review.

We believe that these changes have improved the clarity and readability of our manuscript and hope that it now meets the standards for publication in PLOS ONE. We look forward to your final decision.

Thank you for your valuable input and consideration.

Sincerely,

Naaz Shareef

On behalf of my research team
